# Supplementary material for: CYP3A Excipient-Based Microemulsion Prolongs the Effect of Magnolol on Ischemia Stroke Rats
Source: Pharmaceutics. 2020 Aug 5;12(8):737. doi: 10.3390/pharmaceutics12080737 (PMC7464078; doi:10.3390/pharmaceutics12080737)
Supplement: Supplementary file 1 [file pharmaceutics-12-00737-s001.pdf]

pharmaceutics

Supplementary Materials: CYP3A Excipient-Based Microemulsion Prolongs the Effect of Magnolol on Ischemia Stroke Rats

Jiun-Wen, Guo, Chih-Cheng Chien, and Jiann-Hwa, Chen

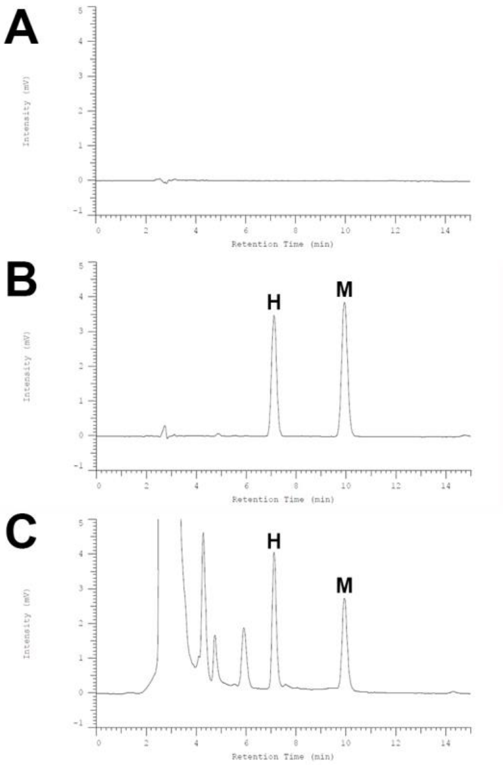

Figure S1. Representative high-performance liquid chromatography of magnolol (M) and honokiol (H). (a) blank (b) standard with magnolol (2μg/ml) and internal standard (honokiol) (c) plasma sample 60 min after 30mg/kg of intraperitoneally administrated magnolol. The retention times of honokiol and magnolol were 7.1 ± 0.2 min and 9.9 ± 0.3 min, respectively. H, honokiol; M, magnolol.

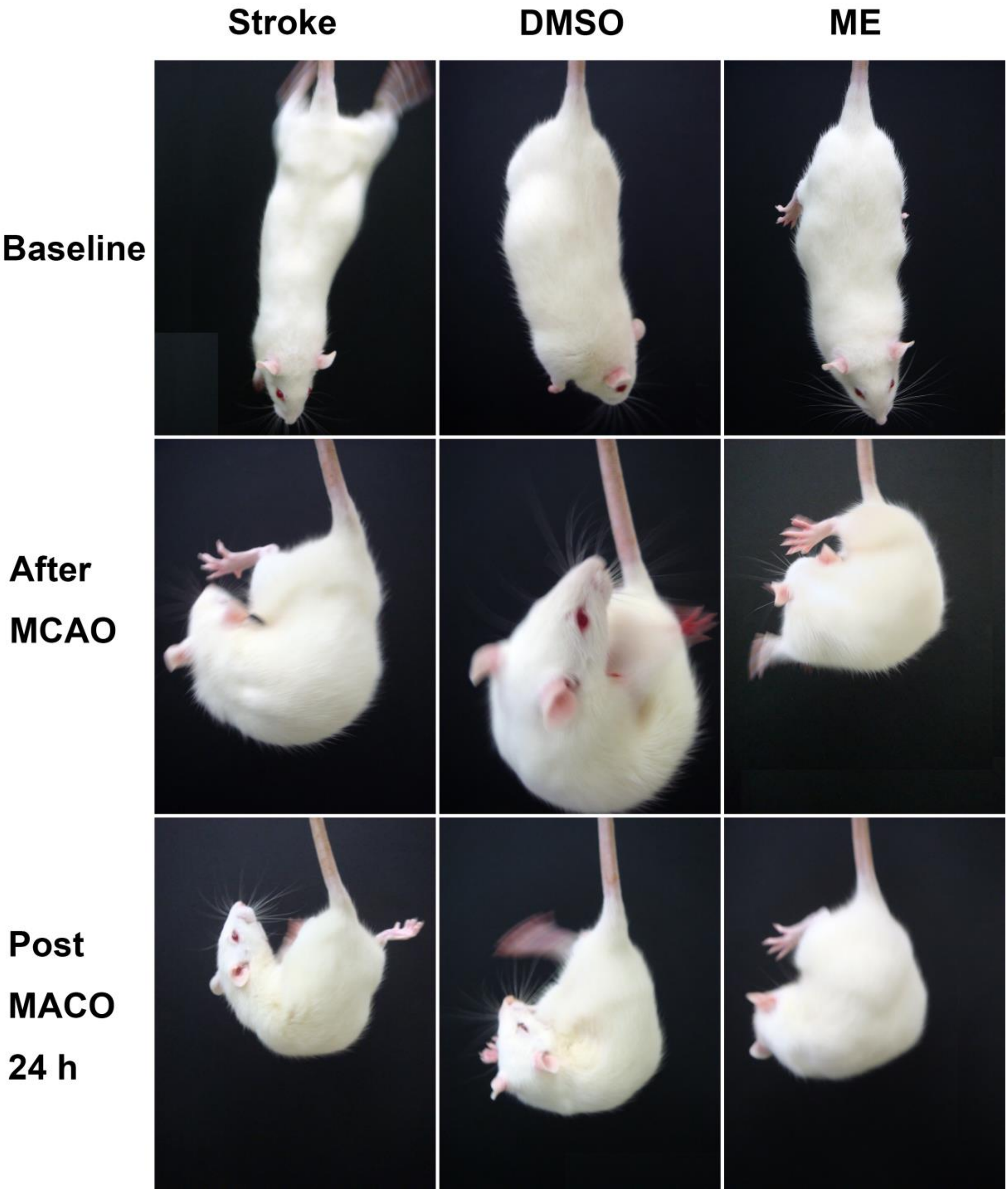

Figure S2. Representative the EBST results of all groups of rats showed C-shaped lateral bending body after MCAO. EBST, elevated body swing test; MCAO, middle cerebral arteries occlusion.

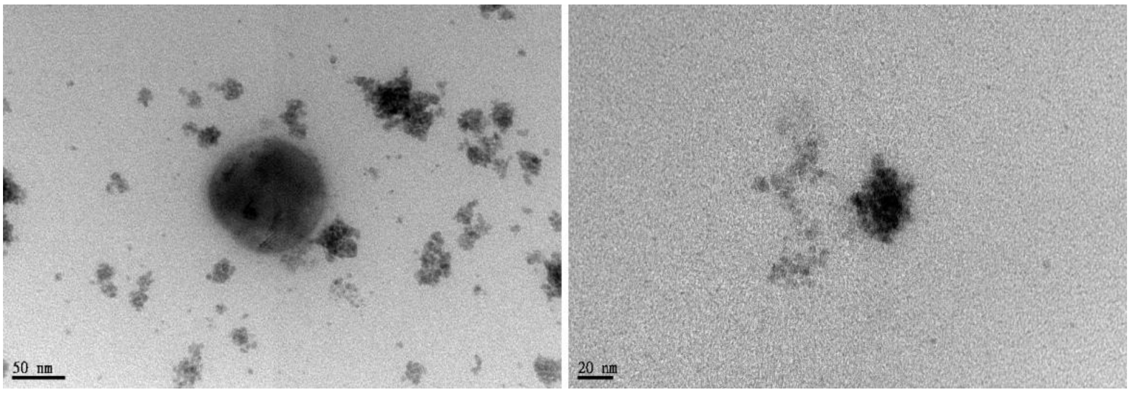

Figure S3. Representative the small particle size micelle of formulation B under the observation by TEM. The composition of formulation B was described in Table 1. TEM, transmission electron microscopy.
